# Evidence-Based Guideline on Critical Patient Transport and Handover to ICU

**DOI:** 10.1155/2021/6618709

**Published:** 2021-05-06

**Authors:** Tesfaye Belaneh Agizew, Henos Enyew Ashagrie, Habtamu Getinet Kassahun, Mamaru Mollalign Temesgen

**Affiliations:** Department of Anesthesia, College of Medicine and Health Sciences, University of Gondar, Gondar, Ethiopia

## Abstract

The perioperative period is a time in which significant physiological change occurs. Improper transfer of information at this point can lead to medical errors. Planning and preparation for critical patient transport to ICU is vital to prevent adverse events. Critical patient transport to ICU must be as safe as possible and should not cause additional risks. It needs good communication, planning, and appropriate staffing with standard monitoring. Evidence shows inconsistency and variability on the use of standardized protocols during critical patient transfer and handover to the ICU. There is a variety of controversial approaches about the need of sedation, use of end-tidal CO_2_ monitoring, and manual versus mechanical ventilation based on different evidence. The objective of this review was to recommend safer options of critical patient transfer to the ICU that help reduce patient morbidity and mortality. *Methods*. Google Scholars, PubMed through HINARI, and other search engines were used to search high-quality evidence that help reach appropriate conclusions. *Discussion*. Critical patient transfer and handover to ICU is a complex procedure that needs experienced hands, availability of appropriate team members, standard monitoring, and necessary emergency and patient-specific medications. Appropriate and adequate transfer of patient information to the receiving team decreases patient morbidity and mortality when the transfer team uses standardized checklist. *Conclusion*. Involvement of senior physicians, use of standard monitoring, and appropriate transfer of information have been shown to decrease critical patient morbidity and mortality.

## 1. Introduction

The postoperative period is a time in which significant physiological change occurs, and this is the time in which the patient recovers from the acute instabilities resulting from anesthesia and surgery. Inadequate and improper transfer of information at this point can lead to medical errors [1]. Transporting patients is a risky procedure, and it requires good communication, planning, and appropriate staffing. Any patient who requires transportation must be effectively stabilized before departure. Planning and preparation are vital to prevent the adverse event [2]. Common postoperative physiologic disorders include nausea and vomiting, oliguria, hypoxia, hypotension, hypothermia, bleeding delirium, pain, and delayed awakening [3].

The main purpose of transporting critically ill patients to the intensive care unit (ICU) is to improve patient prognosis or to reduce morbidity and mortality. Increasing monitoring capabilities are vital during critical patient transportation, and the transport must be as safe as possible and should not cause additional risks [4]. Monitoring battery supply problem, lack of same emergency drug, unplanned intubation, accidental extubation, oxygen supply failure, transport ventilator malfunction, poor transport team, and accidental intravenous line dislodgment may occur on transporting critical patients [5].

One study in France showed that 45.5% of critical patients suffered one or more adverse events during transport to ICU. Risk factors for complication were ventilation with positive end-expiratory pressure >6 cm H2O, sedation before transport, and fluid loading [6]. The incidence of arterial desaturation is highest during this period so that functional pulse oxymetery is mandatory during this time [7].

Patient handover is the transmission of information, professional responsibility, and accountability between individuals and teams [8]. Some study shows that anesthesiologists and ICU nurses had different beliefs concerning the content of information and opinions on what information needs to be reported [9]. The handover and transport of critically ill pediatric patients require communication amongst multiple disciplines. Poor communication is associated with nearly 64% to 70% of sentinel events in the hospital (The Joint Commission, 2015) [10]. Transport and handovers between operating theatres and intensive care departments pose unique challenges [11]. Transfer of critical patients from the operation theatre to ICU is considered to be a high-risk period for the development of vital sign derangement [12].

Guidelines and minimum standards should be available in performing a uniform practice. We commonly encounter controversy on which information, for whom, at what time should be transferred [13].

Currently, some research studies show that large numbers of critically ill patients who encountered significant risks were poorly managed during transport and handover [14]. One study conducted in USA identiﬁed that postoperative handover is informal, unstructured, and inconsistent with often incomplete transfer of essential information [15]. Patient transfer and handover to ICU is extremely complex and is often characterized by incomplete, unstructured, and unsafe practices [16]. Operation theatre to ICU hand offs are known sources of medical error because of poor handover practice [17]. Henceforth, we need to review evidence that focuses on current practices and to recommend safer options of critical patient transfer to the ICU that help reduce patient morbidity and mortality.

## 2. Methodology

A comprehensive search for evidence was conducted at Pub Med/PMC, Google Scholar, and Cochrane database review using appropriate filtering method. Key terms like “critical patient,” “intensive care unit,” “transfer,” “handover,” and “transportation” were used in various combinations.

**Figure 1:**
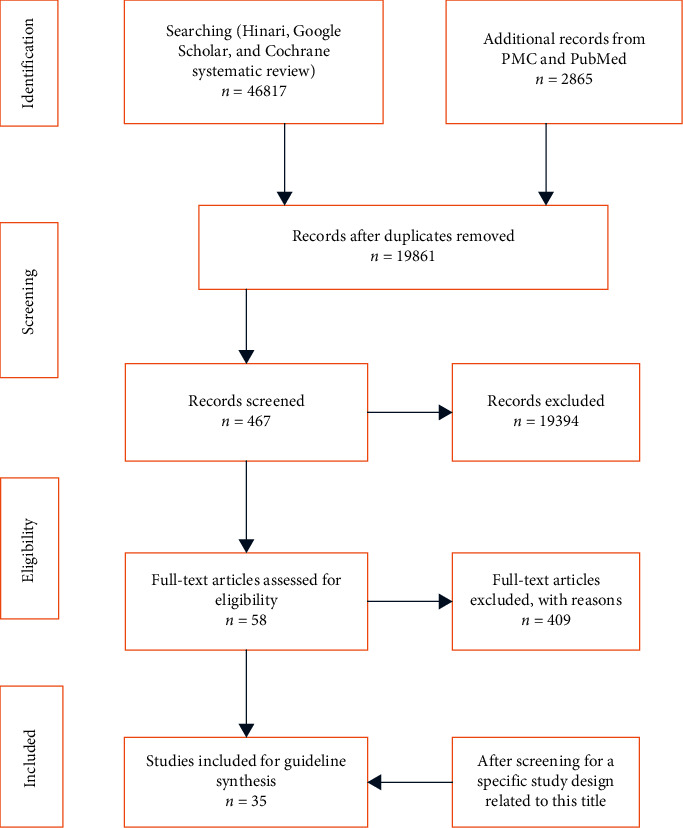
PRISMA 2009 Flow Diagram searching.

After collecting reasonable amount of evidence (Table 1), appraisal of quality of studies by using different institutional appraisal checklists was used to categorize evidence into levels. Final conclusions and recommendations were reached based on the WHO 2011 level of evidence (Table 2). Using PRISMA 2009 flow diagram, searching of systematic reviews, RCTs, evidence-based guidelines, and cohort studies were filtered for the development of guideline (Figure 1).

## 3. Results

One systematic review showed patient transfer and handover was characterized by poor teamwork and communication. The review reported that patients arrive in a compromised state, and there were technical errors, unstructured processes, unclear procedures, interruptions, and distractions during handover. It recommended that all team members should be available, complete urgent task before transfer of information, and allow patient-specific handover and all things must be cleared out [8].

Another systematic review described postoperative handovers as a complex work process challenged by interruptions, time pressure, PACU staff turnover, and a lack of supporting framework. Interventional studies introduced standardized handover tools in combination with environmental changes, resulting in better ﬂow of information, and the main purpose of handover is to create a cognitive picture of the patient that leads to effective decision-making [18].

One systematic review recommended the stabilization, maintaining secured airway, using minimum monitoring, inserting intravenous line, and sedating and paralyzing of intubated patients before transferring. The review added that the patient should be mechanically ventilated, and the team should have in hand of emergency drugs, securing the patient within the transport trolley, and finally filling and signing of the transport form by the consultant [8]. A study conducted at University of Oxford classifies patient handover to ICU into four phases: prehandover, equipment and monitoring handover, information handover, and discussion and plan handover [19]. Studies conducted in France found deficits on information transfer regarding type of anesthesia, type of procedure, duration of surgery, medication history, any intraoperative event, fluid input/output, and postoperative special concern [1].

One systematic review suggests that checklist-centered interventions may be effective in improving the quality and completeness of critical patient handover. Hardwiring within the hospital system through use of uniform tools and methods allow chances to ask questions, reinforce quality and measurement through integration into medical governance and ongoing audit, as well as education and training in the conduct of successful handovers [20].

Patients with lower probability of recovery from anesthesia, those with respiratory failure and need invasive ventilation, patients who require life support for organ failure [21], and patients in of need of intensive monitoring, continuous renal replacement therapies, and invasive hemodynamic monitoring are considered critically ill individuals [22].

Common steps during patient transport includes decision to transport, planning to transport, choice of appropriate transport team, choice of appropriate mode of transport, selection of appropriate monitoring and equipment, prediction of possible complication and management options, and implementing the transport [23]. Guidelines showed that the minimum standards of monitoring required during critical patient transport include appropriate trained and skilled staff, continuous cardiac rhythm (ECG) monitoring, noninvasive blood pressure, oxygen saturation (SaO_2_), end-tidal carbon dioxide (in ventilated patients), and temperature monitoring [24].

Severity of illness, comorbidity and postsurgical status, lack of safety procedural protocol, poor facilities structure, length of transfer, availability of monitoring, poor communication, inadequate training, insufficient staffing, and lack of supervision were associated with complication during critical patient transport [25]. Literatures recommended that oxygen, face mask, self-inflating bag, suction equipment, intubation equipment, difficult airway equipment, syringe with needle, NIBP monitoring, pulse oxymetery, capnography, ECG, and all drugs necessary to manage acute life-threatening medical emergencies should be readily available during transport [23, 26].

One randomized control trial found that the amount of important information transmitted during handover increased from 75% in the control group compared with 87.1% in the study group when they used a standardized checklist [27]. A cohort study conducted in Germany showed that postoperative residual neuromuscular blockade was strongly associated with ICU admission [28]. Handover includes patient information such as name, age, weight, allergies, diagnosis, procedure performed, general condition of the patient, previous medical history, and any coexisting disease [15]. Type of anesthesia, any intraoperative events, medications, IV fluids, estimated blood loss, critical patient monitoring plan, and plan for IV fluids should be handed over appropriately [29]. Surgical site dressings, tubes, drains, and packing; surgical complications; and interventions should be included in the handover phase [30].

The German Association of Anesthesiology and Intensive Care recommends the genuine relationship between organizational prerequisites, professional skills, behaviors, and actions to perform safe transfer patients to ICU [31]. The team consists of surgeon, anesthetist, ICU nurses, and ICU admitting provider participated in transport and handover [29]. Intensive care physician summarizes the postoperative plan in the presence of the ICU team, surgeon, and anesthesiologist [32].

## 4. Conclusion

The transfer and handover of the critically ill patient are not standardized throughout even though some inconsistent guidelines are available. Maintaining adequate sedation and analgesia throughout transport can make the patient calm and comfortable and help avoid unnecessary interruption during transport; however, patient instability and inadequate training of the care provider may increase the risk for interruption [33]. In contrast, a randomized controlled study shows an increased ICU stay and hypoxia in mechanical ventilated patient in sedated patients [34].

Guidelines showed that the minimum standards of monitoring required during critical patient transport include appropriate trained and skilled staff, continuous cardiac rhythm (ECG) monitoring, noninvasive blood pressure, oxygen saturation (SaO_2_), end-tidal carbon dioxide (in ventilated patients), and temperature monitoring [24]. Another study does not recommend routine monitoring of end-tidal CO2 during short transport times in adult patients [35].

Manual ventilation delivers unpredictable tidal volumes [36] and transport ventilators consistently deliver the expected tidal volume, but their battery duration has been questioned [37]. A randomized trial argued the high increase in end-tidal CO_2_ with bag-mask ventilation [38] against another study that showed no significant change from the baseline [39].

A decision to transfer critical patients should be made by senior physicians in the unit after full assessment and communication between referring and the receiving team. Transporting patients is a risky procedure, and it requires good communication, planning, and appropriate staffing. Any patient who requires transportation must be effectively stabilized before departure. Planning and preparation for critical patient transport to ICU are vital to prevent adverse events.

**Figure 2:**
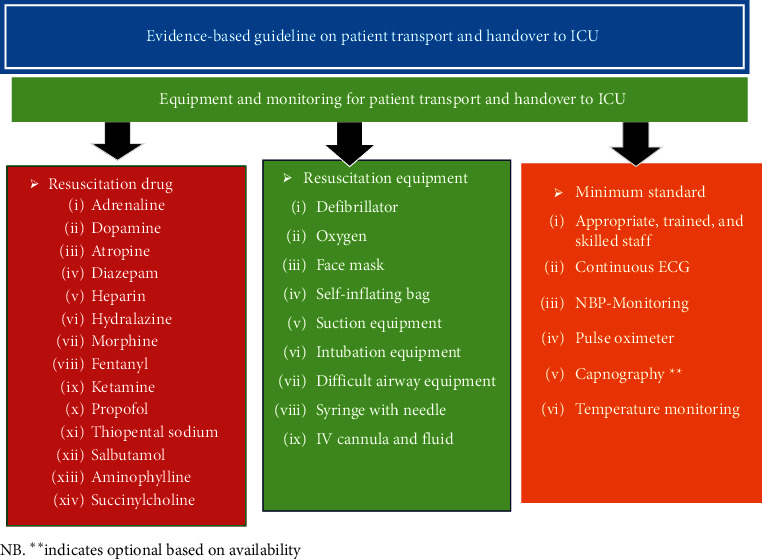
Equipment and monitoring needed for patient transport and hand over to ICU.

**Figure 3:**
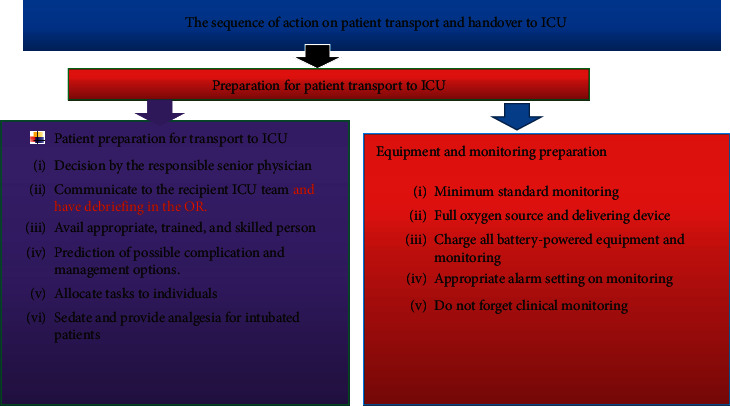
Sequence of activities during critical patient transfer and and over to ICU.

**Figure 4:**
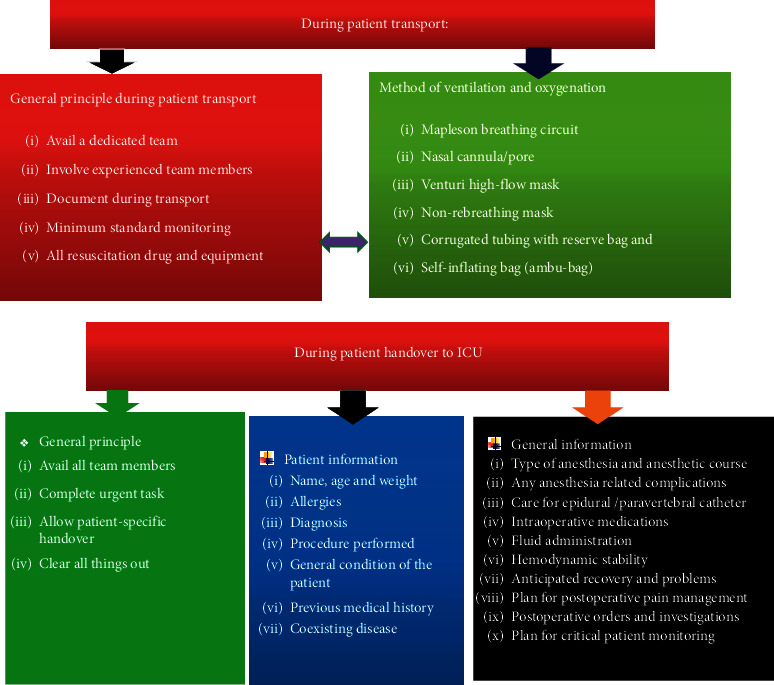
Patient monitoring and mode of ventilation on patient transfer to ICU.

**Figure 5:**
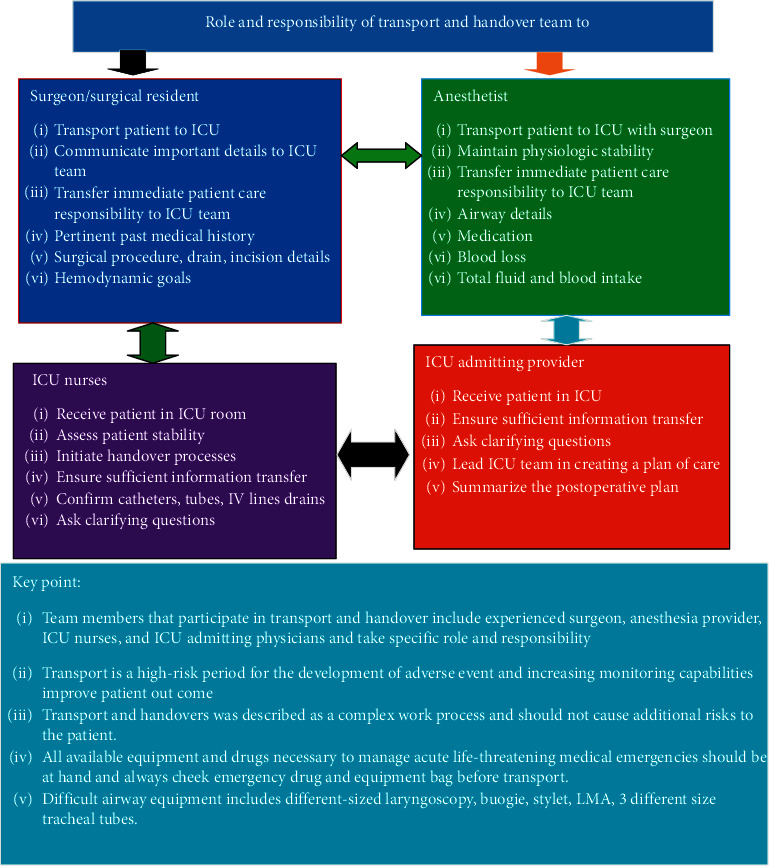
Roles and responsibilities of the critical care givers during patient handover to ICU.

For better patient outcome and safety, use of minimum standard monitoring devices is advisable. Critical patient transport to ICU should be performed early for indicated patients in the postoperative period. Adequate prevention of residual neuromuscular block may decrease rates of unplanned intensive care unit admission. All team members who participate in transport and handover take specific role and responsibility Figures 2–5.

## Figures and Tables

**Table 1:** Summary of the characteristics of included studies.

S.N	Author	Year	Study design	No. of patients/studies	Study intervention	Result/outcome	Recommendation
1.	Segall et. al	2012	Systematic review	31	Transfer and handover to ICU	(i) Urgent task before handover (ii) Resuscitation and stabilization (iii) Allow patient-specific handover (iv) Use minimum standard monitoring (v) All things must be cleared out	Strongly recommended
2.	Møller et. al	2013	Systematic review	23	Postoperative handover	(i) Postoperative handovers are complex work process (ii) Handover leads to effective decision-making (iii) Use standard protocol	Strongly recommended
3.	Robertson et. al	2014	Systematic review	29	Interventions employed to improve intrahospital handover	Information on (i) Postoperative orders and investigations (ii) Critical patient monitoring plan (iii) Plan for IV fluids and (iv) Surgical site complications and interventions.	Recommended
4.	Pucher et. al	2015	Systematic review		Effectiveness of interventions to improve patient handover	(i) SHARE protocol for handover (ii) Standardization of critical patient handover content (iii) Hardwiring within the hospital system through use of uniform tools and methods	Highly recommended
5.	Foronda et. al	2016	Integrated review	40	Handover and transport of critically ill children	(i) Gap in transport and handover (ii) Use of standard communication handoff tool (iii) Communication between the operating room and intensive care staff (iv) Involving specialized teams decreases the morbidity	Recommended
6.	Salzwedel et. al	2016	RCT	134	The effect of a checklist on the quality of patient handover	(i) Checklist increases the quality and quantity of information handover (ii) Increase handover quality from 75% to 85.4% (iii) Check list from the Joint Commission, 2015	Highly recommended
7.	Jayasekera et. al	2015	Guideline		Transport of adult critical care patient	(i) The sequence of action during transport (ii) Appropriate, trained, and skilled staff (iii) Continuous (ECG) monitoring (iv) Noninvasive blood pressure (v) Oxygen saturation (SaO2) (vi) End-tidal carbon dioxide	Highly recommended
8.	Netes et. al	2016	Guideline		Critical patient transfer indication/admission to ICU	(i) Need of intensive care therapies (ii) Need of invasive ventilation (iii) Need of continuous invasive hemodynamic monitoring (iv) Require life support therapy for organ failure (v) Need of intensive monitoring and therapies only provided in the ICU	Highly recommended
9.	New Zealand college of anesthesia	2015	Guideline		Guidelines for the transport of critically ill patients	(i) Sources of oxygen and airway equipment (ii) Difficult airway equipment (iii) Use of standard monitors (iv) Emergency and patient-specific medications	Highly recommended
10.	Knight et. al	2015	Cohort	102	Factors for complication during critical patient transport	(i) Severity of illness, comorbidity and postsurgical status (ii) Lack of safety procedural protocol (iii) Poor facilities structure, length of transfer (iv) Availability of monitoring and equipment (v) Poor communication, inadequate training, insufficient staffing	Recommended
11.	Swickard et. al	2018	Retrospective cohort study	50	Patient safety events during critical care Transport	(i) Adverse event during transport	Recommended
12.	Nagpal et. al	2010	Prospective cohort study	65	Postoperative handover	Required information (i) Name (ii) Age, weight (iii) History of allergies (iv) Diagnosis and procedure performed (v) General condition of the patient (vi) Previous medical history (vii) Any coexisting disease	Recommended

**Table 2:** Levels of evidence and degree of recommendation, Good clinical practice, GCP, WHO, 2011.

Level	Type of evidence	Degree of recommendation
1a	Evidence-based guideline, systematic reviews of RCTs	Strongly recommended/directly applicable
1b	Systematic review	Highly recommended/directly applicable
1c	Randomized clinical trials/RCTs	Recommended/applicable
2a	Systematic reviews of case control or cohort studies.	Extrapolated evidence from other studies
3a	Nonanalytic studies, e.g., case reports and case series	Extrapolated evidence from other studies

Source: Good clinical practice, GCP, WHO, 2011.
